# Supplementary material for: Patterns of Intron Gain and Loss in Fungi
Source: PLoS Biol. 2004 Nov 30;2(12):e422. doi: 10.1371/journal.pbio.0020422 (PMC532390; doi:10.1371/journal.pbio.0020422)
Supplement: Table S1 — Also available at http://genes.mit.edu/NielsenEtAl/. (4.3 MB ZIP). [file pbio.0020422.st001.zip › NielsenEtAl/html/693.html]

AN0647.1.NCU04243.1.MG00977.1.FG09929.1


```
 CLUSTAL W (1.82) Multiple Sequence Alignments - Introns Inserted


Sequence 1: MG00977.1	295 aa
Sequence 2: FG09929.1	298 aa
Sequence 3: NCU04243.1	295 aa
Sequence 4: AN0647.1	282 aa
Alignment Length: 303 aa
Number Identitical Residues: 147 aa
Alignment Score (without introns) 7756


MG00977.1 	MI0RKQARERRDYLYRKVAILKDAEINEKKAKMRASLAQGRPLEKDIANDEKLRADYHYD
NCU04243.1	MI0RKQARQRRDYLYRRALLLKEAEVAEKRAKLRSALASGKPLDPEIAKDKELRKDFDYD
FG09929.1 	MI0RKQARQRRDYLYRRALLLRDAEISEKRAKLRSSLASGKPLDPTIANDKSLRKDYQYD
AN0647.1  	--~---------------MQLRDASIAEKRAQLKESLATGKQLDPSIANDRKLREDFKFD
          	                    *::*.: **:*::: :** *: *:  **:*..** *:.:*

MG00977.1 	ESRIDR-------EDLDLDDEYAQLSGLVDPRVMVTTSRDPSARLAAFAKEIRLLLPTAI
NCU04243.1	VSRDVN------GDAIDIDDEYSELSGVIDPRILVTTSRDPSSRLGQFSKEIRLLLPTSV
FG09929.1 	ESRPDLTT----NEELDLDDEYAQLSGIVDPRVLVTTSRDPSARLAAFAKEIRLLLPTAV
AN0647.1  	ESLPTADKKDKDADLLDLDDEYALTSGIIDPRPIVTTSRSPSVRLGTFAKEIRLLLPTSI
          	 *     ..... : :*:****:  **::*** :*****.** **. *:*********::

MG00977.1 	RLNRGNLILPELVRSVQAAGLSDIVLLHERRGTPTALTISHM~PHGPTVSFSLHNVLLRH
NCU04243.1	RLNRGNLVLEDLVGAAKAQNLTDVVLLHEHRGVPTAMTISHF0PHGPTLMVSLHNVVLRA
FG09929.1 	RLNRGNLILPDLVRSAQSAGLSDVILLHEHRGTPTALTLSHF~PHGPTVSFSLHNVVLRH
AN0647.1  	RLNRGNLVLPDLVASANSASLTDMVILHEHRGTPTAMTISHL~PHGPTASFSLHNVVLRA
          	*******:* :** :.:: .*:*:::***:**.***:*:**: *****  .*****:** 

MG00977.1 	DIPGSVRGTVSEAYPHLVFDGFTSKLGQRIVKVLKHLFPPREPITNKAKMSNRVITFKNI
NCU04243.1	DIPKSIKGTVSESYPRLIFEGFSTKLGERVVKILKHLFPPREP-TQKPNVGNRVITFVNN
FG09929.1 	DIPGSVRGTVSESYPHLIFDGFTSRLGERIVKILKHVFPPREAITSKNKVGNRVVTFKNI
AN0647.1  	DIPNAARGTVSESYPHLIFEGFKTKLGKRVVQILKHLFPPREA----GKVGNRVVTFVNK
          	*** : :*****:**:*:*:**.::**:*:*::***:*****.     ::.***:** * 

MG00977.1 	DDVIEVRHHVFVRTGHESVELAEVGPRMSMKVFEIRGGTLEN-KDGDVEWHLSQYTRTSR
NCU04243.1	DDTIEVRHHVYVRTSYDSVELSEVGPRFTMKPFKITMGTLDN-KDADTEWHLSQYTRTSR
FG09929.1 	EDSIEVRHHVFVRTGYDSVELAEVGPRMTMRPFEIRSGTLEN-KDGDVEWHLTQYTRTAK
AN0647.1  	EDSIEVRHHVFVKTGYKDAELAEVGPRMTMRLFEIRGGSLEKGSSGDVEWALTQYTRTSR
          	:* *******:*:*.:...**:*****::*: *:*  *:*::....*.** *:*****::

MG00977.1 	KKNYL
NCU04243.1	KKNYF
FG09929.1 	KKNYL
AN0647.1  	KKDYL
          	**:*:
```
